# Whispers of the Lungs: Unraveling the Mystery of Catamenial Pneumothorax

**DOI:** 10.7759/cureus.94210

**Published:** 2025-10-09

**Authors:** Mang Ning Ong, Kean Leong Koay, Ananbabu Palaniappan, Muhammad Ishamuddin Ismail, Mohd Ramzisham Abdul Rahman

**Affiliations:** 1 Department of Surgery, Universiti Kebangsaan Malaysia Medical Centre, Kuala Lumpur, MYS; 2 Department of Surgery, Cardiothoracic Surgery Unit, Universiti Kebangsaan Malaysia Medical Centre, Kuala Lumpur, MYS

**Keywords:** cardiothoracic surgery, catamenial, catamenial pneumothorax, endometriosis, gynecology, pneumothorax, spontaneous pneumothorax, thoracic endometriosis syndrome, vats, video-assisted thoracoscopic surgery (vats)

## Abstract

Catamenial pneumothorax (CP) is an uncommon condition marked by recurrent spontaneous pneumothorax in women, typically occurring within the first 72 hours of menstruation. It is also the most prevalent manifestation of thoracic endometriosis syndrome. Although the exact pathophysiology of CP remains unclear, several hypotheses have been suggested. Despite its rarity, CP should be suspected in women presenting with recurrent spontaneous pneumothorax, particularly if episodes align with menstrual cycles. Early recognition and multidisciplinary management involving thoracic surgeons and gynecologists are essential for optimizing outcomes. This case highlights the importance of considering CP as a differential diagnosis in female patients with recurrent pneumothorax and reinforces the role of video-assisted thoracoscopic surgery and hormonal therapy in management.

## Introduction

Pneumothorax is defined as the abnormal presence of air within the pleural space, resulting in impaired lung expansion and respiratory distress. It may arise spontaneously, following trauma, or as a complication of medical procedures. Spontaneous pneumothorax can be primary, occurring without pre-existing lung disease, or secondary, when linked to underlying pathologies such as chronic obstructive pulmonary disease, infections, or systemic disorders.

Catamenial pneumothorax (CP) is an uncommon condition characterized by recurrent spontaneous pneumothorax in women, typically occurring within the first 72 hours of menstruation. It is estimated to account for approximately 3-6% of spontaneous pneumothorax cases in menstruating women [1]. However, some studies suggest that CP may represent a higher percentage, ranging from 18% to 33% of spontaneous pneumothorax cases in women [2]. This variation in reported incidence may be attributed to underdiagnosis and limited disease awareness.

CP is also the most prevalent manifestation of thoracic endometriosis syndrome (TES), which includes other conditions such as catamenial hemothorax, catamenial hemoptysis, and pulmonary nodules associated with endometriosis [1]. The underlying mechanisms of CP remain debated, with several theories proposed. These include the movement of endometrial tissue through small defects in the diaphragm following retrograde menstruation, the spread of endometrial cells via blood or lymphatic circulation, and the transformation of pleural or diaphragmatic cells through coelomic metaplasia. Despite these explanations, no single theory fully accounts for all cases [1].

Diagnosing CP and TES can be difficult, as the presentation often resembles that of spontaneous pneumothorax, and imaging findings may be inconspicuous. When recognition is delayed, patients are at risk of repeated episodes and increased morbidity. Therefore, greater clinical awareness is essential, as early identification and appropriate surgical or medical treatment can help prevent recurrence and improve outcomes.

Here, we report the case of a 40-year-old woman with recurrent right-sided pneumothorax whose symptoms coincided with her menstrual cycle. Through clinical evaluation, imaging, and operative findings, a diagnosis of CP was established.

## Case presentation

A 40-year-old nulliparous woman with no significant medical or surgical history was referred to the cardiothoracic team for recurrent pneumothorax. She initially reported intermittent episodes of shortness of breath with right-sided pleuritic chest pain over the past two years, typically coinciding with her menstrual cycle. The patient reported regular menstrual cycles with moderate flow with occasional dysmenorrhea. She had not been previously evaluated for endometriosis and had no prior gynecological interventions. The symptoms usually resolved when her cycle ended, with the onset occurring within 24 to 72 hours after the start of menstruation. Over time, her symptoms progressively worsened, and a chest radiograph (CXR) confirmed a spontaneous right apical pneumothorax. She was admitted and managed conservatively with a high-flow mask. A follow-up CXR showed resolution of the pneumothorax, and she was discharged without residual symptoms.

**Figure 1:**
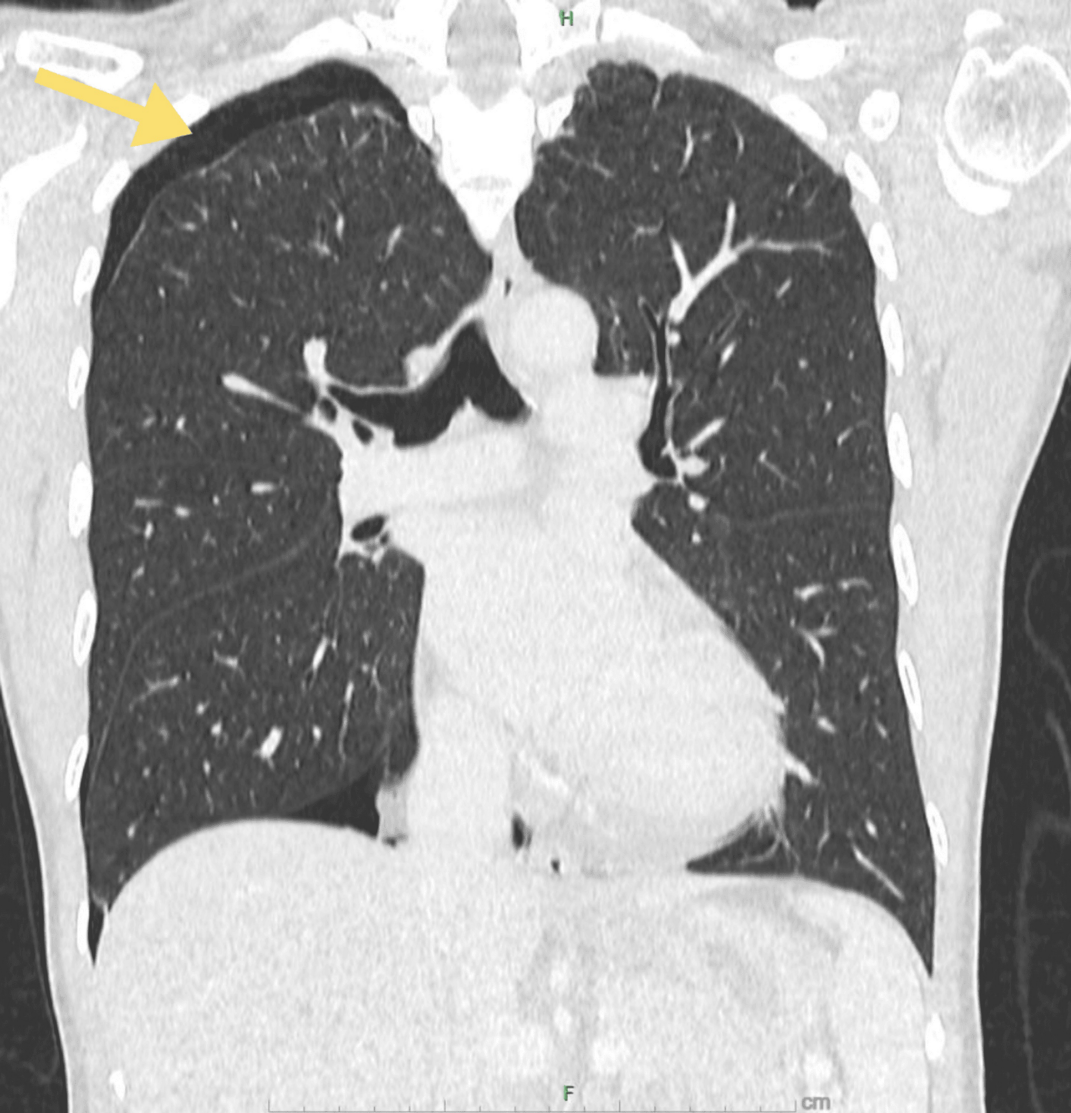
CT of the thorax (coronal view). The image shows a right apical pneumothorax (shown by the yellow arrow).

One month later, she presented with recurrent right-sided pleuritic chest pain. A CT scan of the thorax revealed a small pneumothorax (1.4 cm in width) at the right apex (Figure 1). No significant ground-glass opacities or infective changes were noted. Given the recurrent nature of her condition, the possibility of CP was considered. She was again managed conservatively.

**Figure 2:**
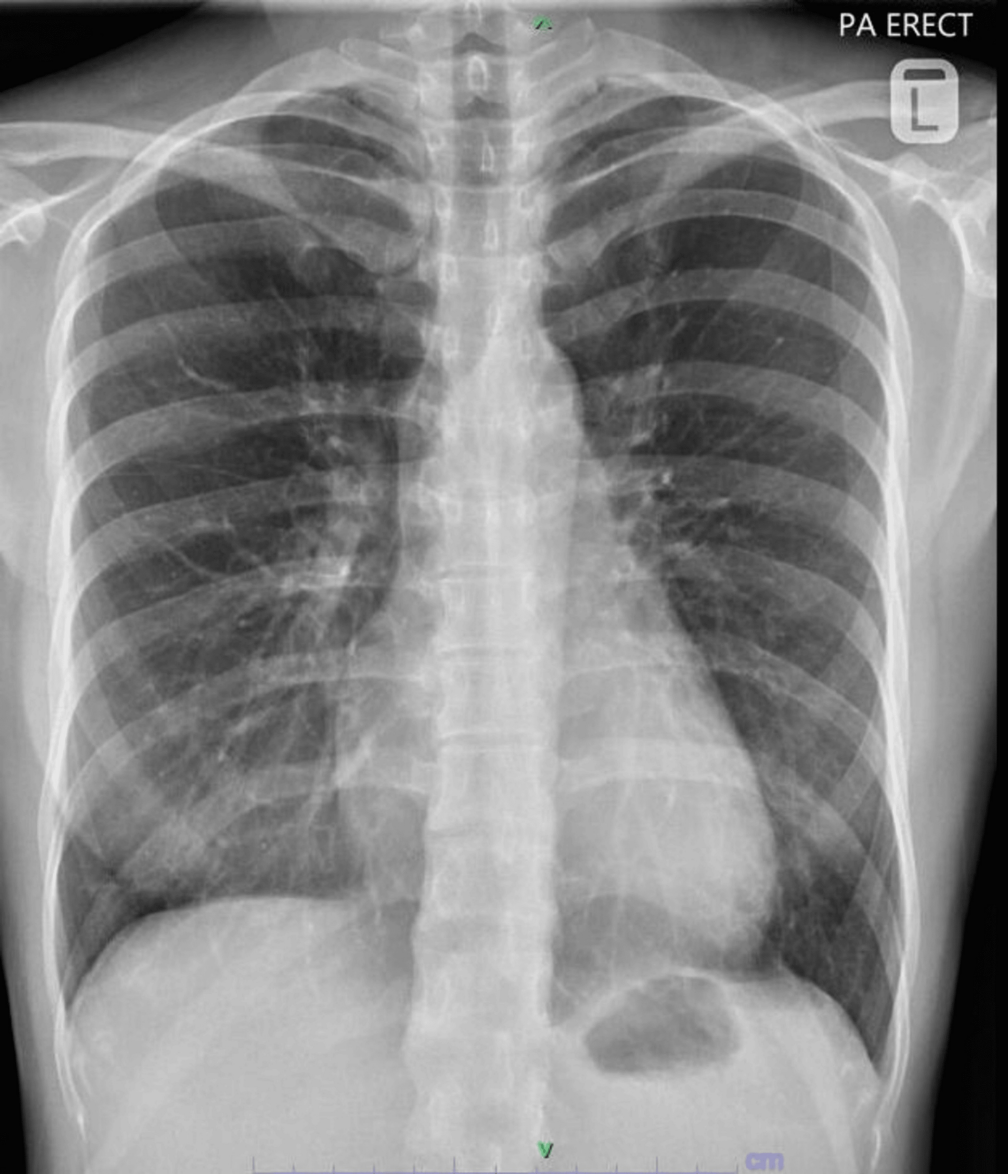
Chest radiograph (posteroanterior erect). The image obtained preoperatively shows the resolution of pneumothorax.

The patient was subsequently referred to our center for further evaluation and management. Physical examination at the time of presentation was unremarkable, and laboratory studies (Table 1) revealed no abnormalities. A repeat CXR at our center showed resolution of the pneumothorax (Figure 2). She was scheduled for an elective right video-assisted thoracoscopic surgery (VATS) with wedge resection and pleurodesis.

**Table 1:** Laboratory results. ALT: alanine aminotransferase; AST: aspartate aminotransferase; ALP: alkaline phosphatase; PT: prothrombin time; INR: international normalized ratio; APTT: activated partial thromboplastin time

Parameters	Results	Range	Unit
Hemoglobin	12	12.0–15.0	g/dL
Platelet	319	150–410	10^9^/L
White cell count	4.8	4–10	10^9^/L
Urea	3.7	2.5–6.7	mmol/L
Sodium	138	136–145	mmol/L
Potassium	4.5	3.5–5.1	mmol/L
Creatinine	62	49–90	µmol/L
ALT	9	0–55	U/L
ALP	59	40–150	U/L
AST	20	5–34	U/L
ALP	46	35–50	g/L
Total protein	80	64–83	g/L
Total bilirubin	6.2	3.4–20.5	µmol/L
PT	12.9	11.6–14.9	Seconds
INR	0.93	-	-
APTT	30.5	30.3–46.5	Seconds

**Figure 3:**
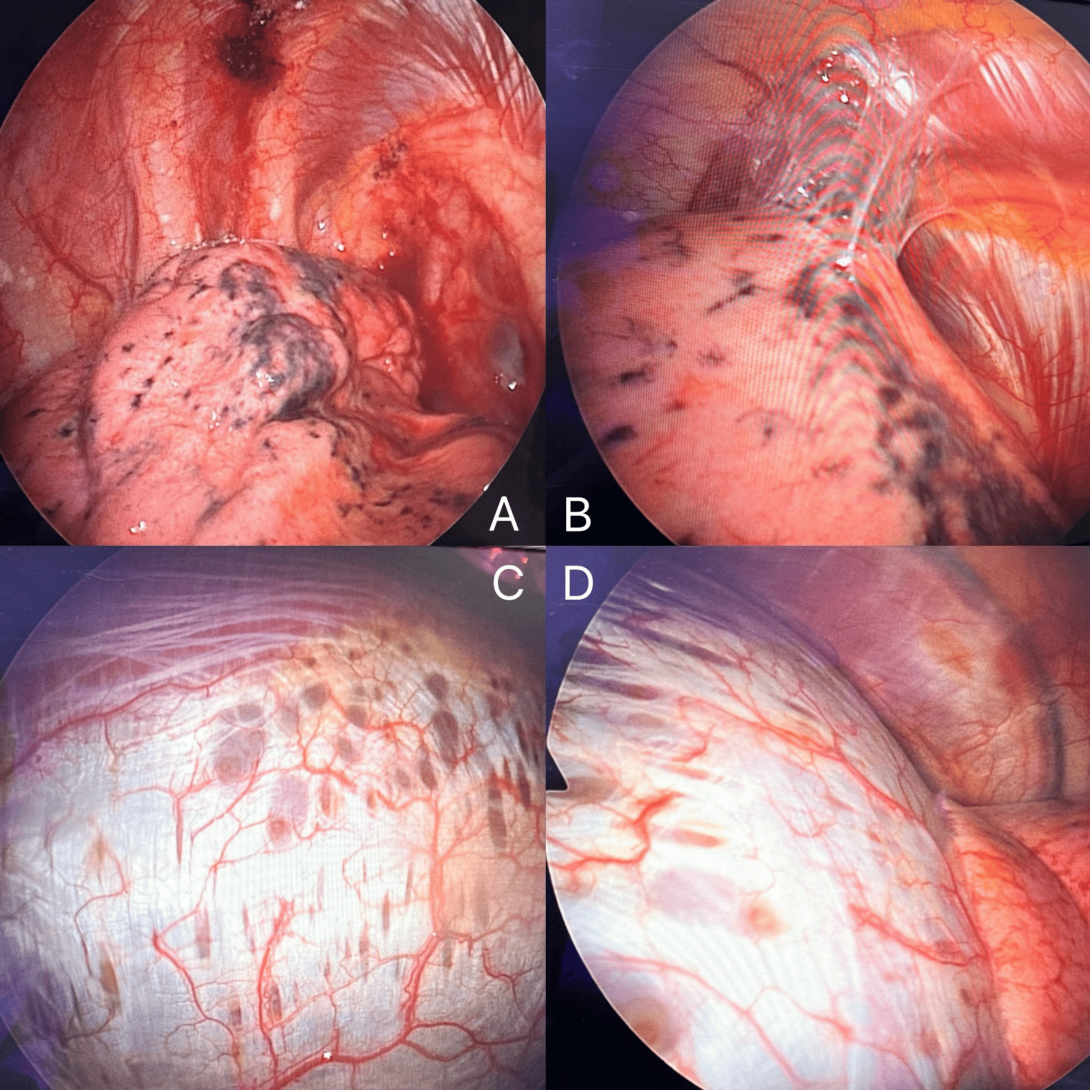
Intraoperative findings. (A, B) Adhesions at the apical region of the pleura, thickened and bluish parenchymal suggestive of multiple endometriotic lesions over the right upper and middle lobes. (C, D) Characteristic diaphragmatic lesions in a patient with catamenial pneumothorax. Multiple circular spots and fenestrations at the dome of the right diaphragm. Diaphragmatic fenestrations are small defects in the diaphragm that can serve as pathways for air or endometrial tissue to pass from the peritoneal to the pleural cavity. Their presence supports the theory of transdiaphragmatic passage as a mechanism of catamenial pneumothorax and is considered a characteristic intraoperative finding in thoracic endometriosis.

Intraoperative findings revealed adhesions at the apical region of the pleura, with thickened bluish tissue at the apex of the right lung, which was identified as the source of recurrent pneumothorax. Multiple endometriotic lesions were observed over the right upper and middle lobes, as well as numerous endometriotic spots on the diaphragm. A wedge resection was performed at the apex, and multiple enlarged endometriotic spots were removed. The central diaphragm appeared thin and exhibited multiple fenestrations, making direct suturing and plication unfeasible (Figure 3). An endomesh was placed over the endometriotic spots over the diaphragm and tacked into position. Surgical and medical pleurodesis with gentamicin and talc was also performed. These intraoperative findings were consistent with thoracic endometriosis, thereby confirming the diagnosis of CP.

Following surgery, the patient was referred to a gynecologist for further management of her endometriosis. Hormonal therapy was recommended to reduce the risk of recurrence, with gonadotropin-releasing hormone (GnRH) analogs and oral contraceptive pills suggested. She was planned for three cycles of GnRH analogue (Dipherelin 3.75 mg), followed by Visanne 2 mg daily until she decided to conceive. Postoperative recovery was uneventful, and she was discharged on postoperative day five.

**Figure 4:**
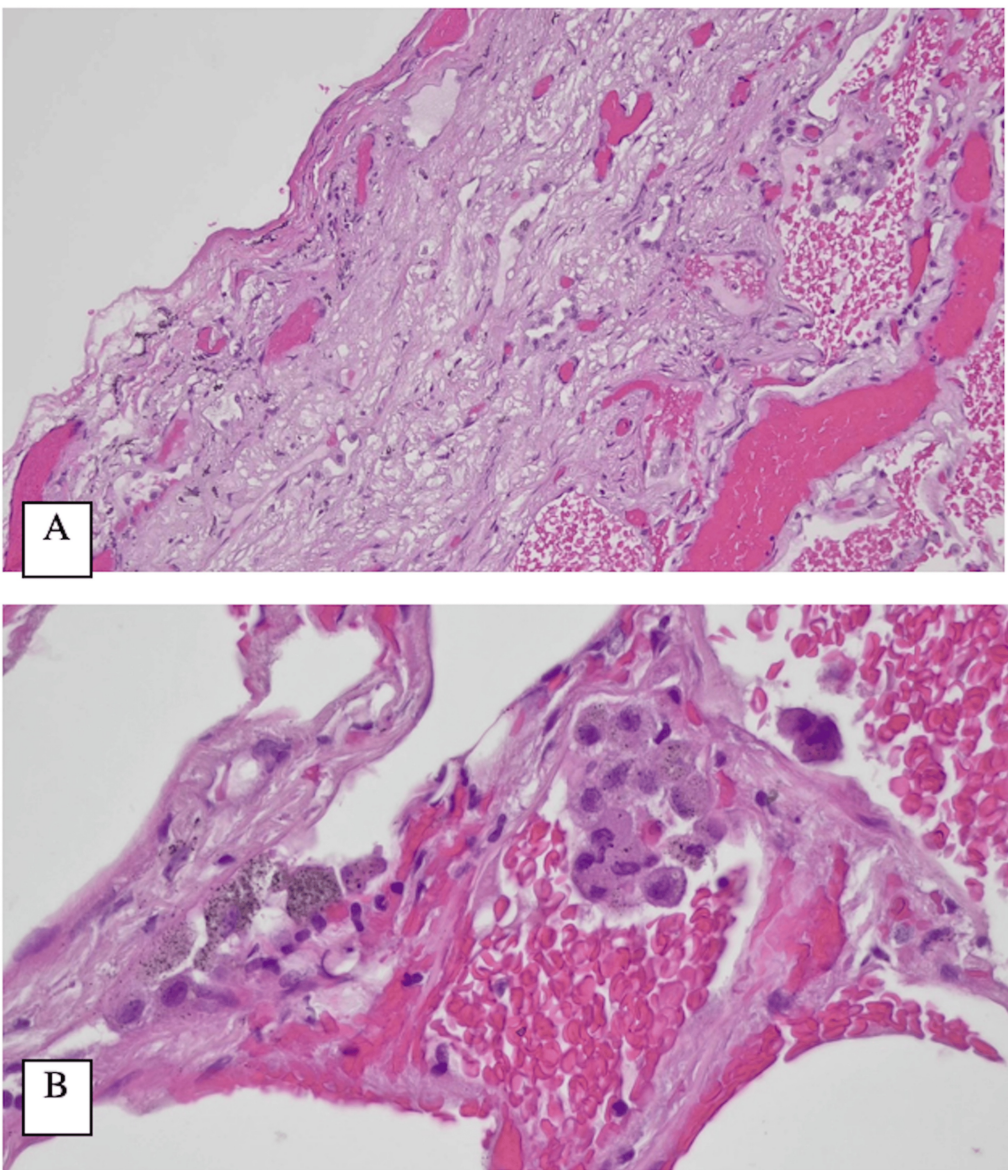
Photomicrographs of the right lung upper lobe biopsy. (A) Subpleural fibrosis (hematoxylin and eosin, 200×). (B) Foci of hemorrhage with collections of pigment-laden macrophages (hematoxylin and eosin, 600×).

During subsequent follow-ups at the cardiothoracic outpatient clinic, the patient remained well. Histopathological examination of the resected lung tissue revealed irregularly sized and dilated air spaces with fragmented alveolar walls, mild fibrosis, and infiltration by lymphocytes and plasma cells (Figure 4). However, no endometrial tissue was identified. Immunohistochemical studies showed that estrogen receptor staining was negative.

Despite the absence of direct histological evidence of endometrial tissue, the clinical presentation and intraoperative findings remained highly suggestive of thoracic endometriosis.

## Discussion

TES is an under-recognized condition characterized by the presence of ectopic endometrial tissue within the thoracic cavity [3]. TES encompasses several clinical manifestations, including CP, catamenial hemothorax, catamenial hemoptysis, and pulmonary nodules [1]. Among these, CP is the most prevalent, accounting for approximately 73% of TES cases [4].

CP is a rare condition characterized by spontaneous, recurrent pneumothorax in women of reproductive age, typically occurring within 24-72 hours of menstruation onset, and predominantly affecting the right lung. The term “catamenial” originates from the Greek word “katamenios,” which signifies a monthly occurrence [1]. It is estimated to account for approximately 3-6% of spontaneous pneumothorax cases in menstruating women [1]. However, some studies suggest that CP may account for a higher percentage, ranging from 18% to 33% of spontaneous pneumothorax cases in women [2].

Although the exact pathophysiology of CP remains unclear, several hypotheses have been suggested. A widely accepted theory involves the movement of air or endometrial tissue from the peritoneal cavity into the pleural space through diaphragmatic defects, which are commonly found in affected individuals [5,6]. Another possible mechanism is the presence of ectopic endometrial implants within the pleura, which undergo cyclical hormonal changes, leading to localized inflammation, tissue breakdown, and air leakage during menstruation [4]. Additionally, prostaglandin-mediated alveolar rupture has been proposed, where hormonal fluctuations trigger bronchospasm and increased intrathoracic pressure, ultimately resulting in alveolar damage and pneumothorax [7]. Although no single explanation fully accounts for all cases, these mechanisms likely work in conjunction, contributing to the recurrent nature of CP in affected women.

The diagnosis of CP is challenging, due to its cyclical nature and non-specific symptoms, and it is often misdiagnosed as primary spontaneous pneumothorax. Imaging studies, including CXR and CT of the thorax, may reveal pneumothorax, diaphragmatic defects, or pleural abnormalities. VATS is the gold standard, as it allows direct visualization and biopsy of endometriotic lesions [4]. Histopathological confirmation of endometrial tissue within the pleura further supports the diagnosis.

However, a negative biopsy does not exclude CP. It is important to note that, in our case, histopathological examination did not reveal ectopic endometrial tissue. Histology may often be negative due to the small size or patchy distribution of lesions, missed sampling, or other technical limitations. Further, a considerable proportion of clinically and surgically confirmed cases of CP lack histological confirmation [5,6]. In such circumstances, clinical correlation with imaging findings, intraoperative observations, and symptom patterns remains crucial for diagnosis.

The management of CP typically involves a combination of surgical and hormonal therapies aimed at reducing recurrence. VATS is the preferred surgical approach, allowing for the repair of diaphragmatic defects, pleurectomy, pleurodesis, and the excision of ectopic endometrial lesions [4,8]. Studies have reported VATS success rates ranging from 81.2% to 100%, with some cases demonstrating recurrence rates as low as 0% over follow-up periods of up to 36 months [9].

Adjunctive hormonal therapy, including GnRH analogs, progestins, and estrogen-progestin combinations, is commonly used to reduce the risk of recurrence in CP. These therapies are typically administered continuously or for 3 to 18 months. Few studies have shown that combining minimally invasive surgical techniques with hormonal treatment leads to lower recurrence rates compared to surgery alone [8,10]. This highlights the importance of addressing both the structural abnormalities through VATS and the hormonal factors contributing to disease progression, making this dual approach an effective strategy for managing CP and thoracic endometriosis.

## Conclusions

CP, the most prevalent form of TES, is often underdiagnosed despite its cyclical recurrence linked to menstruation. Clinical awareness is crucial, especially for women of reproductive age with cyclic spontaneous pneumothorax and recurrent pleuritic chest pain. While histological confirmation is ideal, negative pathology does not exclude the diagnosis, as clinical history and intraoperative findings frequently provide stronger diagnostic clues. Accurate diagnosis requires thorough evaluation through imaging studies and VATS, which facilitates both diagnosis and targeted treatment. A multidisciplinary approach, combining surgical intervention and hormonal therapy, is essential to prevent recurrence and improve outcomes. Enhanced clinician awareness and further research into the pathophysiology of CP will aid in early diagnosis and better therapeutic strategies, ultimately improving patient quality of life.
